# Perforator-based propeller flaps treating loss of substance in the lower limb

**DOI:** 10.1007/s10195-011-0136-0

**Published:** 2011-05-05

**Authors:** Pierluigi Tos, Marco Innocenti, Stefano Artiaco, Andrea Antonini, Luca Delcroix, Stefano Geuna, Bruno Battiston

**Affiliations:** 1 Department of Orthopedics and Traumatology, UOD Reconstructive Microsurgery, CTO-M. Adelaide, Turin Via Zuretti 29, 10126 Turin, Italy; 2 Department of Orthopaedics, Traumatology, Rehabilitation, Plastic and Reconstructive Sciences, Second University of Naples, Naples, Italy; 3 UO Reconstructive Microsurgery, CTO, Florence, Italy; 4 Department of Clinical and Biological Sciences, University of Turin, Turin, Italy

**Keywords:** Perforator local flaps, Propeller flaps, Lower-limb reconstruction

## Abstract

**Background:**

Local flaps based on perforator vessels are raising interest in reconstructive surgery of the limbs. These flaps allow efficient coverage of large wounds without the need to sacrifice a major vascular axis. The operative technique does not require microvascular anastomosis and allows reconstruction of soft tissue defects using nearby similar tissues. The aim of this study was to evaluate the clinical results of local perforator flaps in the treatment of complex lower-limb defects.

**Materials and methods:**

Twenty-two local perforator flaps were retrospectively studied. Loss of substance was due to postsurgical complications in seven cases, oncological resection in six, posttraumatic defect in five, pressure sores in three, and osteomyelitis in one.

**Results:**

Postoperatively, two patients showed partial flap necrosis. In five patients, a superficial epidermolysis occurred. Minor complications were seen in three patients who showed transient venous congestion of the flap. Furthermore, transient leg edema was sometimes observed in patients with large propeller flaps. All but one patient healed without further major surgical procedures. In three cases, secondary skin grafts were performed. In most cases, the aesthetic result was optimal and patients were fully satisfied.

**Conclusions:**

When characteristics of the defect are suitable for treatment with a propeller-based local flap, this technique should be considered as one of reasonable options for surgical reconstruction. Microsurgical techniques facilitate the management of complex trauma in emergency and may allow planning reconstructive procedures and limb salvage in elective orthopedic surgery.

## Introduction

During the last few years, the strategy for treatment of lower-limb soft tissue defects has changed due to the introduction of new models of local flaps. Improvement in the anatomical knowledge on cutaneous, subcutaneous, and intramuscular vessels originating from major vascular axis of the limbs [1–3] has allowed development of several types of perforator flaps, which today are commonly employed in clinical practice. According to the Gent consensus [4], perforator flaps are constituted by areas of cutaneous and subcutaneous tissue nourished by perforator branches originating from deep vascular axis with an intramuscular [musculocutaneous perforator flap (MCPF)] or intraseptal [septocutaneous perforator flap (SCPF)] course. In coverage by means of V–Y advancement, the local perforator-based flap reaches remarkable distances superior to those obtained with standard V–Y flap [5]. In the harvesting method by means of pedicle torsion, the local perforator-based flap is isolated and rotated around the perforator branch as a propeller for a maximum angle of 180° [6], according to the original concept that Hyakusoku introduced to treat burn-scar contractures [7]. Blades of the propeller flap differ in dimensions and can be designed, according to defect feature, in a “freestyle” technique following the origin and direction to the cutaneous paddle of the perforator vessels [8]. Although local flap technique requires microsurgical dissection, it does not require vascular suturing and can thus be defined a microsurgical nonmicrovascular flap as reported by Georgescu et al. [9]. Avoiding vascular sutures makes the surgical act quicker in comparison with microvascular flaps, and the pedicle can be skeletonized under loops magnification and not necessarily under microscope. Yet, major vascular axes with surrounding muscles are preserved, reducing donor-site morbidity. From the aesthetic point of view, deficit reconstruction leads to optimal results because the like-with-like reconstruction concept is respected due to employment of donor-tissue areas located near the defect. All these advantages contribute to the continuously increasing use of local perforator flaps in reconstructive microsurgery of both upper and lower limbs in cases of simple and complex loss of substance. So far, the largest clinical trial was reported by Georgescu et al. [9] for treating forearm and hand substance defects. Clinical applications have also been described for trunk, head, neck, and perineal region reconstruction [10, 11]. As far as the lower limb is concerned, a single perforator vessel may nourish a large fasciocutaneous area, even in sites considered unreachable or at risk for local flaps as the inferior third of the leg and ankle. As reported by Teo, main perforators arise in the leg from the posterior tibial, peroneal, and anterior tibial artery. The first two vessels are the easier ones on which the flap can be based [6]. The peripatellar region can also be covered by means of propeller flaps elevated from the distal anteromedial aspect of the thigh and nourished by perforator branches of the saphenous, femoral, and descending genicular artery [12]. Therefore, the propeller flaps may be employed in lower-limb reconstruction for a wide spectrum of clinical application, including posttraumatic defects, oncological resections, and postoperative wound dehiscence such as those that occur after fracture management, Achilles tendon surgery, and prosthetic knee replacement. The aim of this study was to evaluate clinical results of local perforator-based propeller flaps in treating complex lower-limb defects. We present a retrospective analysis of propeller flaps performed in a group of patients affected by loss of substance of the lower limb operated in our departments. This is one of the largest and most detailed case series of propeller flaps performed in reconstructive microsurgery units.

## Materials and methods

### Case series

**Fig. 1:**
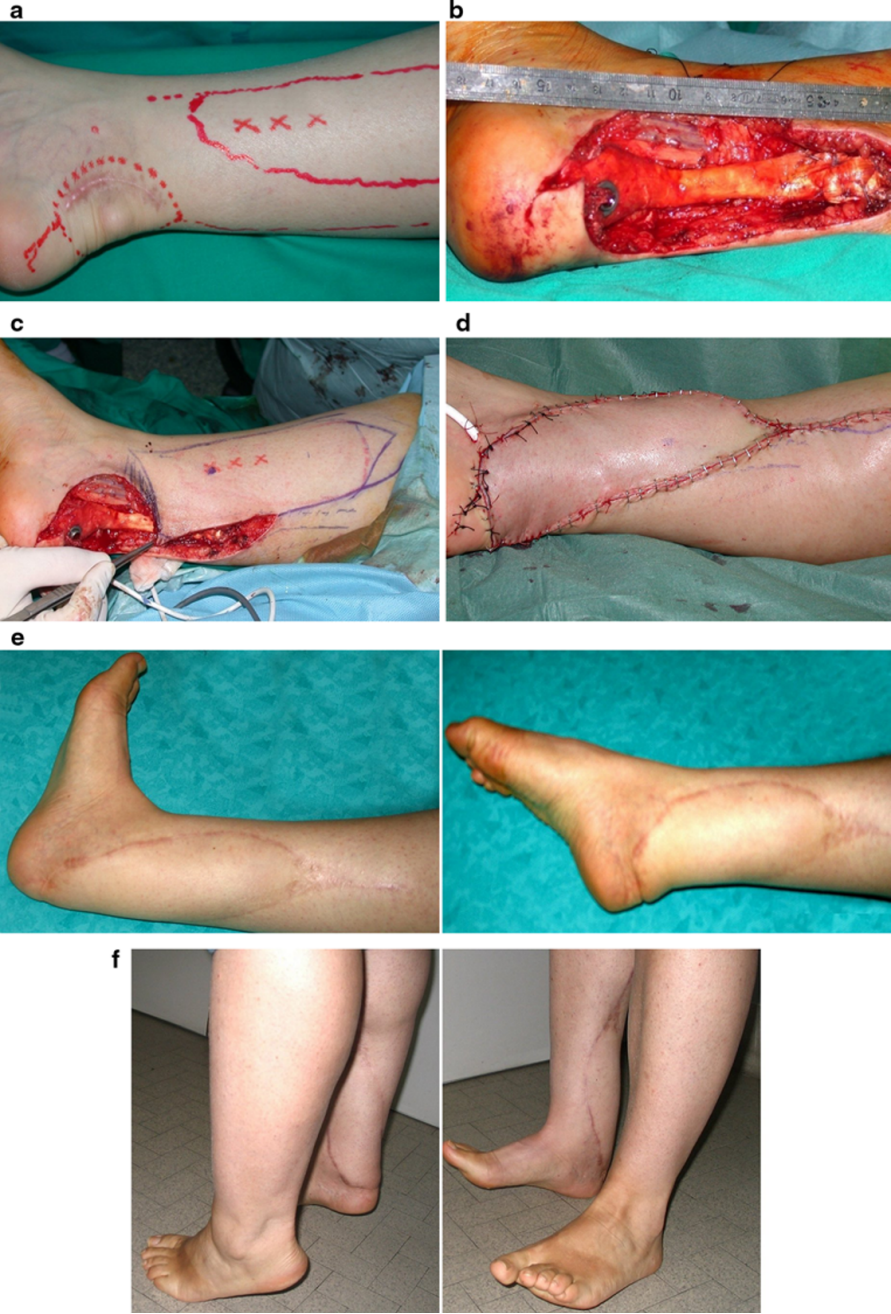
Ectopic chondrosarcoma Achilles tendon. **a** Preoperative planning on a flap based on posterior tibial artery perforators. **b** Intraoperative defect after surgical excision and Achilles tendon reconstruction with an allograft. **c** New surgical planning after excision. **d** Postoperative view with the propeller flap turned around the perforator vessel 170° and direct closure. **e, f** Aesthetic and functional result (follow-up 6 months) (Case 2, Table 1)

Case series involved 22 patients operated on over a period of 4 years. All patients treated in our departments in this period with propeller flaps were included in the study. No patients were lost to follow-up. All patients gave their informed consent prior to being included. The study was performed according to the ethical standards of the 1964 Declaration of Helsinki as revised in 2000. There were 11 women and 11 men and the mean age at the time of surgery was 56.5 (range 22–86) years. Etiology of the loss of substance was postsurgical wound defect in seven cases (five Achilles tendon repair; one femoral bypass; one knee arthrolysis), oncological resection in six cases (five soft tissue sarcomas, one chondrosarcoma), posttraumatic defect in five cases (lower-limb open fractures), pressure sore in three cases, and chronic osteomyelitis in one case. Before the surgical procedure, two patients received a nonoperative treatment consisting of wound care and vacuum-assisted closure (VAC) therapy to improve the status of the soft tissue bed in the recipient area. The defect was located at the leg or ankle in 17 cases, foot in five, distal third of the thigh in one, and groin in one. In one case, after resection of an ectopic chondrosarcoma located in the Achilles tendon, reconstruction required the use of an Achilles tendon allograft before soft tissue coverage by means of a posterior tibial artery perforator-based propeller flap (Fig. 1a–d). Table 1 summarizes characteristics and size of the defect for all patients, the originating perforator artery, the size of the flap, and the degree of rotation.

**Table 1:** Case series

Case	Age (years)/sex	Etiology loss of substance associate disease	Perforator flap	Flap size (cm)/add graft	Defect size (cm)	Pedicle rotation	Location	Complications treatment
1	70/F	Oncological resection	PA (SC)	3 × 10	2 × 5	100°	Achilles region	Venous congestion
2	22/F	Oncological resection (ectopic chondrosarcoma Achilles tendon)	PTA (SC)	20 × 6/Achilles tendon allograft	9 × 12	170°	Achilles region	
3	86/F	Oncological resection (leiomyosarcoma)	GA (MC)	25 × 12	14 × 20	160°	Distal third thigh	Flap necrosis (50%) secondary skin graft
4	70/M	Postoperative complication (Achilles tendon repair) diabetes	PA (SC)	15 × 4/skin graft	10 × 4	120°	Achilles region	Venous congestion supeficial epidermolysis
5	49/M	Postoperative complication (Achilles tendon repair) diabetes	PTA (SC)	12 × 4/skin graft	15 × 4	150°	Achilles region	Superficial epidermolysis secondary skin graft
6	76/M	Oncological resection (liposarcoma)	PTA (MC)	10 × 22/skin graft	15 × 12	170°	Distal third leg	
7	35/M	Posttraumatic defect (open leg fracture)	PTA (SC)	12 × 5	5 × 3	170°	Distal third leg	
8	38/M	Postoperative complication (Achilles tendon repair)	PA (SC)	4 × 9/skin graft	11 × 4	170°	Achilles region	
9	72/F	Chronic osteomyelitis	PTA (SC)	8 × 14	6 × 4	100°	Distal third leg	Superficial epidermolysis
10	43/M	Postoperative complication	PTA (SC)	12 × 5	5 × 4	80°	Middle third leg	
11	65/F	Oncological resection	PTA (SC)	25 × 15	10 × 15	80°	Distal third leg	
12	67/F	Pressure sore	PA (SC)	3 × 7	3 × 3	140°	Achilles region	Superficial epidermolysis
13	79/M	Posttraumatic defect (open leg fracture)	PTA (SC)	6 × 10	3 × 5	160°	Middle/distal third leg	Flap necrosis (80%)
14	79/M	Postoperative complication (femoral artery bypass)	LCFA (SC)	15 × 25	15 × 10	90°	Groin	
15	64/F	Postoperative complication (Achilles tendon repair)	PTA (SC)	4 × 11	4 × 3	180°	Achilles region	
16	26/M	Posttraumatic defect (open midfoot fracture)	PA (SC)	5 × 12	3 × 4	180°	Foot	
17	23/M	Posttraumatic defect (open ankle fracture)	PTA (SC)	5 × 10	5 × 5	100°	Ankle	
18	68/F	Pressure sore diabetes	PA (SC)	3 × 5	3 × 2	180°	Distal third leg	Venous congestion
19	34/F	Postoncological resection (liposarcoma)	PTA (SC)	6 × 13/skin graft	5 × 6	180°	Foot	
20	72/F	Posttraumatic defect (open calcaneus fracture)	PTA (SC)	5 × 9	3 × 3	180°	Heel	Superficial epidermolysis
21	62/F	Pressure sore diabetes	PTA (SC)	6 × 1/skin graft	5 × 4	180°	Heel	
22	43/M	Postoperative. complication (knee arthrolysis-Judet)	DFA (MC)	9 × 18	5 × 8	90°	Knee	

*PA* peroneal artery, *PTA* posterior tibial artery, *GA* genicular artery, *LCFA* lateral circumflex femoral artery, *DFA* deep femoral artery, *SC* septocutaneous, *MC* musculocutaneous

### Surgical technique

**Fig. 2:**
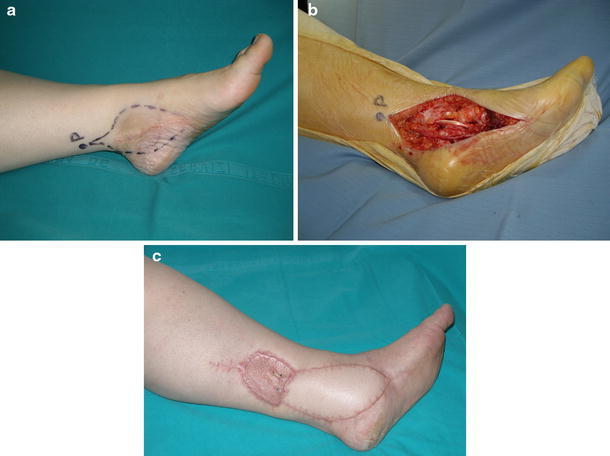
Widening of liposarcoma excission. **a** Preoperative planning and drawing (*P*) of the perforator of the posterior tibial artery. **b** Intraoperative defect after surgical excision. **c** Aesthetic and functional result (follow-up 6 months) (case 19, Table 1)

A handled ultrasound Doppler scanner was used preoperatively to detect perforator arteries in the donor-site area [13]. We adopted a color Doppler duplex drawing on the skin at the points of perforating vessel emergence on the fascia. On this basis, the flap was planned according to the position and size of the defect, taking into account the need to avoid excessive tension on the border of the propeller flap during suturing. When the procedure was performed in emergency, an explorative incision was made to find a perforator artery suitable to harvest a free-style perforator flap. Operations were performed using magnification loupes (2.5–4.0×) and microsurgical instruments, with a careful blunt dissection. An explorative incision, usually through a subfascial approach, was made to directly visualize the perforator vessels. Perforator artery selection before flap harvesting was based on vessel size and distance to the area of the defect. The perforator arteries selected were septocutaneous (SC) in 19 cases and musculocutaneous in three. The flap was then designed centering movement of propeller blades around the point at which the perforator artery emerged from muscle or fascia. Inclusion of a fascia into the flap depended on the donor site and the characteristics of the lesion (bone exposure). During the dissection procedure, particular attention was paid to preserve the vascular pedicle. The pedicle was dissected in a blunt way isolating the perforator branch for a length of 1.5 cm at least, and pedicle traction during flap harvesting and positioning was carefully avoided. Perfusion was checked before flap rotation by waiting a few minutes and irrigating the shin paddle with lukewarm saline solution in order to promote microcirculation recovery. After propeller rotation, the minor skin paddle helped the closure of the largest part of the donor site. If possible, direct closure of the donor site was performed without tension on the edge of the flap. In six patients, skin grafts were required to cover the secondary defect, as direct closure was not possible (Fig. 2a–c). Carefully positioned drains were then applied at the end of the procedure. Drains were usually removed after 24 h. Bandaging was soft, to avoid compression over the flap, and the limb was held in an elevated position. A window was left uncovered to control skin color and temperature without bandage removal. When closure was performed using a skin graft, immobilization for 10 days was prescribed. Low molecular weight heparin was administered only when the limb was immobilized or when the patient was not allowed to walk.

## Results

Detailed description of outcome results and complications is reported in Table 1. Extensive flap necrosis (80% of the surface) was observed in only one patient (case 13). Another patient showed partial necrosis of the free-style perforator thigh flap involving 50% of the surface (case 3) and another five patients had a limited superficial epidermolysis for venous congestion that resolved spontaneously (cases 4, 5, 9, 12, 20). In three patients (cases 3, 5, 13), a secondary skin graft was required to treat complications 1 month after primary reconstruction. In one of these patients (case 13), VAC therapy was used for 2 weeks before skin graft application. Spontaneous healing occurred in the remaining cases with epidermolysis. As for minor complications, three patients showed transient venous congestion of the flap. Transient edema of the affected limb was sometimes recognized. Prolonged leg edema (6 months) with spontaneous resolution was observed in a patient with a large propeller flap covering an Achilles tendon allograft and disappeared in 6 months with the use of compressive stockings, and good functional and aesthetic result was obtained (Fig. 1e, f).

## Discussion

The ideal reconstruction technique for both simple and complex defects of the lower limb should replace like-to-like tissue, minimize donor-site morbidity, preserve main vascular trunks, and reduce operating and hospitalization time. In carefully selected cases, propeller perforator-based local flaps can meet these requirements. The development of propeller flaps in reconstructive microsurgery has been facilitated by improved knowledge of the arterial basis of flap perfusion and anatomical studies on lower-limb vascularization provided the basis for local perforator flap design in treating thigh and leg defect [1, 2, 12–15]. The subdermic vascular network is particularly rich and allows the harvesting of thin skin flaps. One single perforator vessel located in an eccentric position in relation to a skin paddle may support a large skin area thanks to the opening of potential vascular territories, which move to the peripheral border of the flap. The process of vascular adoption is promoted by the increase of blood pressure, which occurs in the perforator artery after closure of subcutaneous and intramuscular branches during flap harvesting. One of the main characteristics of perforator flaps is their versatility, as the flap may be selected on the perforator artery according to defect type and harvested either in free or local form. As it is a local flap, the perforator-based cutaneous paddle may cover the defect through direct advancement or through torsion of the vascular pedicle. The principles for lower-limb reconstruction with perforator local flap have been meticulously described step-by-step by Teo [6]. In particular, this author pointed out the importance of these flaps in covering medial and lateral malleolar areas and of the heel and Achilles tendon. Defects in these anatomical areas, although often small, are usually difficult to treat with alternative nonperforator local flaps. The value of perforator local flaps is further increased by the optimal quality of tissues transferred for defect reconstruction. In contrast, it has been recently observed [16] that in the lower third of the leg and in the ankle region, the inadequate length of the perforator and the presence of tendons may interfere with flap transposition.

Over the last 3 years, several clinical studies reported on the application and results of propeller perforator-based local flaps in lower-limb reconstruction [17–21]. Masia et al. [17] used propeller flaps in 35 of 59 patient operated on with perforator flaps for defects related to oncological surgery, trauma, and unstable scars. They reported four unspecified flap losses and observed partial necrosis with secondary healing in four propeller flaps performed in heavy smokers (three) or diabetic (one) patients. Jakubietz et al. [18] treated eight patients with defects in the malleolar region with 180° propeller flaps based on perforators from the tibial and peroneal vessels. Also, in this case series, a partial flap loss was encountered in an insulin-dependent diabetic patient, whereas partial superficial epidermolysis was observed in two cases and healed without further intervention. Transient leg edema was observed in all patients. Finally, Pignatti et al. [19] described six patients with defects located in knee, tibia, and Achilles tendon areas. In two cases, a transient venous congestion was observed and resolved spontaneously. No flap necrosis was registered in the two latter case series.

The main limitations of our study were that it was retrospective and involved heterogeneous sites. Moreover, the procedures were planned and performed by different surgeons due to the multicentric characteristic of our research. In our clinical experience, perforator-based propeller flaps accomplished preoperative expectations. All patients healed, and no further surgical procedure was required except for secondary skin grafts in three patients. In most cases, the aesthetic result was very good, and patients were fully satisfied. Postoperative complications were observed in nine of 22 patients and in three of four diabetic patients, indicating this disease as the most important risk factor for flap complication. Transient leg edema was sometimes observed in cases of large propeller flaps. The propeller perforator-based local flaps used in the reconstruction of relatively small loss-of-substance cases induced minor donor-site morbidity and led to good aesthetic results because of the use of like-to-like tissues. Operative time was reduced, and no specific medical therapy was required. A potential risk of this intervention is flap failure, which may involve, in case of extensive loss, an amount of propeller larger than the area of the previously untreated defect. This event cannot be underestimated and supports the view that specific dissection training and adequate microsurgical skill are required before performing this tricky procedure. Perforator flaps may represent good alternatives to free flaps in body areas in which local reconstructive procedures are not possible. The favorable results reported in the literature, as well as the results of our personal experience for lower-limb reconstruction, are encouraging. We believe that when the characteristics of the defect are suitable for treatment with a propeller-based local flap, this technique should be regarded as one of the possible reconstructive options. On the other hand, free flaps remain the first-choice solution for covering wide cutaneous areas and complex reconstruction requiring composite or functional flaps. Microsurgery may play a key role in treating orthopedic and trauma patients, and the need for knowledge of microsurgical techniques is growing. Indeed, microsurgery facilitates the management of complex trauma in emergency and may allow planning of reconstructive procedures and limb-salvage operations in elective orthopedic surgery.
